# Hematopoietic stem cell aging: a review of transcriptional and multi-omics insights and potential paths for AI integration

**DOI:** 10.1038/s12276-026-01805-0

**Published:** 2026-08-19

**Authors:** Bongsoo Park, Hagai Yanai, Jun Ding, Isabel Beerman

**Affiliations:** 1 Genetic and Epigenetic Mechanisms of Stem Cells Unit, Translational Gerontology Branch, National Institute on Aging, National Institutes of Health, Baltimore, MD USA; 2 Human Statistical Genetics Unit, Translational Gerontology Branch, National Institute on Aging, National Institutes of Health, Baltimore, MD USA

**Keywords:** Haematopoietic stem cells, Computational platforms and environments

## Abstract

Hematopoietic stem cell (HSC) aging underlies age-related immune decline, anemia and increased risk of hematologic malignancies, including clonal hematopoiesis and leukemia. Many available microarray and bulk RNA sequencing studies have elucidated conserved transcriptional hallmarks, such as myeloid bias, inflammation dysregulation, and self-renewal reinforcement in aged HSCs across mouse and human models. Here, we review key publicly available transcriptome and epigenome datasets from landmark studies, highlighting their contributions to defining HSC molecular aging signatures. We also summarize recent single-cell RNA sequencing, HSC aging intervention, and sex difference datasets. Despite these advances in technology and available sequencing datasets, fragmented data access, limited cross-species integration, and scarcity of multi-omics and single-cell contexts hinder progress. We discuss strategies for dataset harmonization, incorporation of multi-omics (transcriptome, epigenome, and proteome) and single-cell resolution to uncover heterogeneity and trajectories, as well as introduce the application of artificial intelligence and machine learning for predictive modeling, epigenome aging clocks, variant calling, clonal hematopoiesis detection, chromatin-based age prediction, and trajectory inference. Bridging insights from genetic mutant mouse models to emerging human bone marrow organoids offers translational potential for modeling HSC aging in vitro. We propose a curated, centralized, interactive database as a community resource to integrate these layers, enabling meta-analyses, artificial intelligence-driven discoveries, and accelerated therapeutic interventions for age-related hematopoietic disorders.

## Aging hematopoietic stem cells reveal immune system decline

Hematopoietic stem cells (HSCs) are crucial for lifelong blood production, balancing self-renewal, and differentiation. This Review explores the aging of HSCs, highlighting intrinsic defects such as telomere shortening and mitochondrial dysfunction and extrinsic changes in the bone marrow niche. These factors contribute to immunosenescence and leukemia risk. The authors discuss the evolution from microarray to RNA sequencing, revealing transcriptional changes in aged HSCs. Key findings include myeloid bias and stress-primed subpopulations. This Review emphasizes the significance of integrating multi-omics data and artificial intelligence-driven approaches to enhance understanding and potential interventions. Future directions point towards artificial intelligence-enhanced data integration and translational research to accelerate discoveries in HSC aging and rejuvenation strategies. "This summary was initially drafted using artificial intelligence, then revised and fact-checked by the author."

## Introduction

**Fig. 1:**
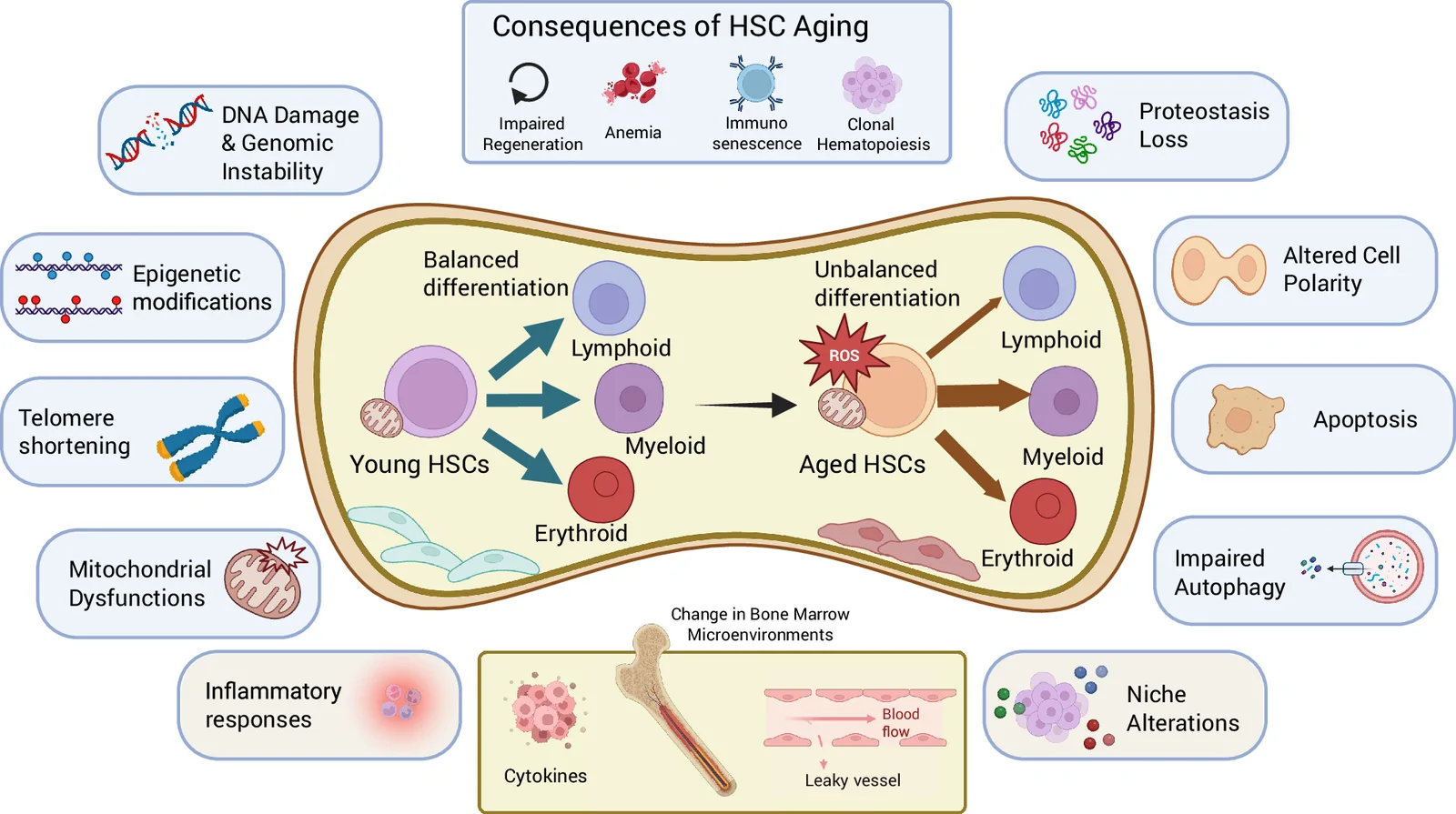
Hallmarks and consequences of hematopoietic stem cell aging. This schematic illustrates the transition from young to aged hematopoietic stem cells (HSCs) and the range of alterations associated with this process. Young HSCs exhibit balanced differentiation into lymphoid, myeloid, and erythroid lineages, whereas aged HSCs demonstrate unbalanced differentiation with a pronounced myeloid bias and increased reactive oxygen species (ROS). The aging process is driven by multiple cellular hallmarks, such as DNA damage, genomic instability, and epigenetic modifications. Extrinsic factors such as niche alterations (changes in bone marrow microenvironments, cytokine profiles, and vascular integrity) and inflammatory responses contribute to HSC decline.

Hematopoietic stem cells (HSCs) sustain lifelong blood production through a balance of self-renewal and differentiation^1,2^. Residing largely in the bone marrow, HSCs generate all blood cell lineages, including immune cells that undergo rapid turnover, so maintaining these differentiated cells is critical for sustained immune homeostasis^3–5^. Figure 1 is a schematic overview of reported intrinsic and extrinsic changes driving HSC aging and their functional outcomes^2–5^. Beyond transcriptional dysregulation, aged HSCs accumulate intrinsic defects including telomere shortening^6^, mitochondrial dysfunction^7^, elevated reactive oxygen species^8^, proteostasis loss^9^, impaired autophagy^10^, and altered cell polarity^11^, alongside extrinsic changes in the bone marrow niche such as altered cytokine signaling^12^ and vascular leakiness^13^ (Fig. 1). These changes contribute to immunosenescence, anemia, and leukemia predisposition in the older adult population in the USA and globally.

Early molecular investigations into HSC aging relied on microarray-based gene expression profiling or DNA methylation arrays, laying out the groundwork for understanding molecular dysregulation. Rossi et al.^14^ provided evidence of cell-intrinsic changes, with aged HSCs showing upregulated genes associated with myeloid specification, stress responses, and leukemia-related pathways, alongside downregulated lymphoid associated genes. Similarly, Chambers et al.^15^ analyzed more than 14,000 genes in highly purified HSCs, identifying approximately 1500 age-induced and 1600 age-repressed transcripts enriched for stress response, inflammation, and epigenetic modifiers, highlighting deficits in preservation functions despite increased stem cell numbers^15^. Norddahl et al.^7^ also revealed that accumulating mitochondrial DNA mutations could drive premature hematopoietic aging phenotypes molecularly distinct from physiologic stem cell aging via additional microarray datasets.

Building on these and other foundational microarray studies, the transition to bulk RNA sequencing (RNA-seq) enabled higher resolution and more quantitative insights into HSC aging transcriptional hallmarks. Bulk RNA-seq studies^5^ demonstrated the upregulation of self-renewal and rRNA-processing genes and began to introduce additional epigenetics analysis at differentiation loci, confirming molecular underpinnings of myeloid bias^16^. Subsequent work uncovered heterogeneous inflammatory responses and stress-primed subpopulations in aged HSCs^4^, whereas human studies linked enhancer reprogramming to oncogenic pathway activation and leukemia risk^3^.

In parallel, early single-cell RNA-seq (scRNA-seq) for HSC aging studies used a SMART-seq-based approach^17,18^, which requires the isolation of HSC cells into plates and performing single-cell sequencing per isolated well. Additional approaches — 10×-based transcriptomics^19^, joint multi-omics profiling (for example, ATAC-seq, DNA methylation, and proteomics), and machine-learning classifications — have further illuminated intra-HSC heterogeneity, clonal trajectories, and niche crosstalk. Meanwhile, human iPSC-derived bone marrow organoids are emerging as scalable in vitro platforms for modeling HSC aging^20^, niche interactions, and genetic perturbations in a human context. However, datasets based on bulk cell samples generating transcriptome and epigenome data using high-resolution genome-wide epigenetic profiling methods also have been expanding the volume of high-resolution transcriptome, epigenome, and 3D topological data available^5,21^. Despite these advances, foundational bulk RNA-seq datasets (and their microarray predecessors) remain scattered across repositories, complicating cross-study meta-analyses and multilayer integration with scRNA-seq datasets.

In addition to these developments and challenges, we are witnessing a profound shift in scientific discovery driven by artificial intelligence (AI), particularly in biology. For instance, an AI model trained on continuous glucose monitoring data outperforms the standard biomarker HbA1c in predicting incident type 2 diabetes and cardiovascular mortality^22^. AI systems have been developed for various applications and applied in collaborative scientific networks^23^. This trend is also reflected in the rapid increase in journals (for example, *Nature Machine Intelligence*), publications, conferences, and workshops dedicated to AI applications in biology from 2024 to the present. Further cooperation between molecular and computational biologists facilitated by AI is poised to shape foundational directions for the advancement of molecular biology and genomics.

In this Review, we summarize key foundational studies on HSC aging signatures and propose future directions in epigenetics and stem cell aging research. AI-driven approaches to database integration and translational research will be central to future breakthroughs, markedly accelerating the pace of discovery in the coming years. We also discuss the current limitations of AI in the context of epigenetics and stem cell biology. Finally, we highlight the latest discoveries in HSC aging mechanisms, discuss the challenges of elucidating molecular mechanisms using experimentally controlled mouse models, and explore their translational potential for human populations.

## Key transcriptional and epigenomic profiling datasets on HSC aging

**Fig. 2:**
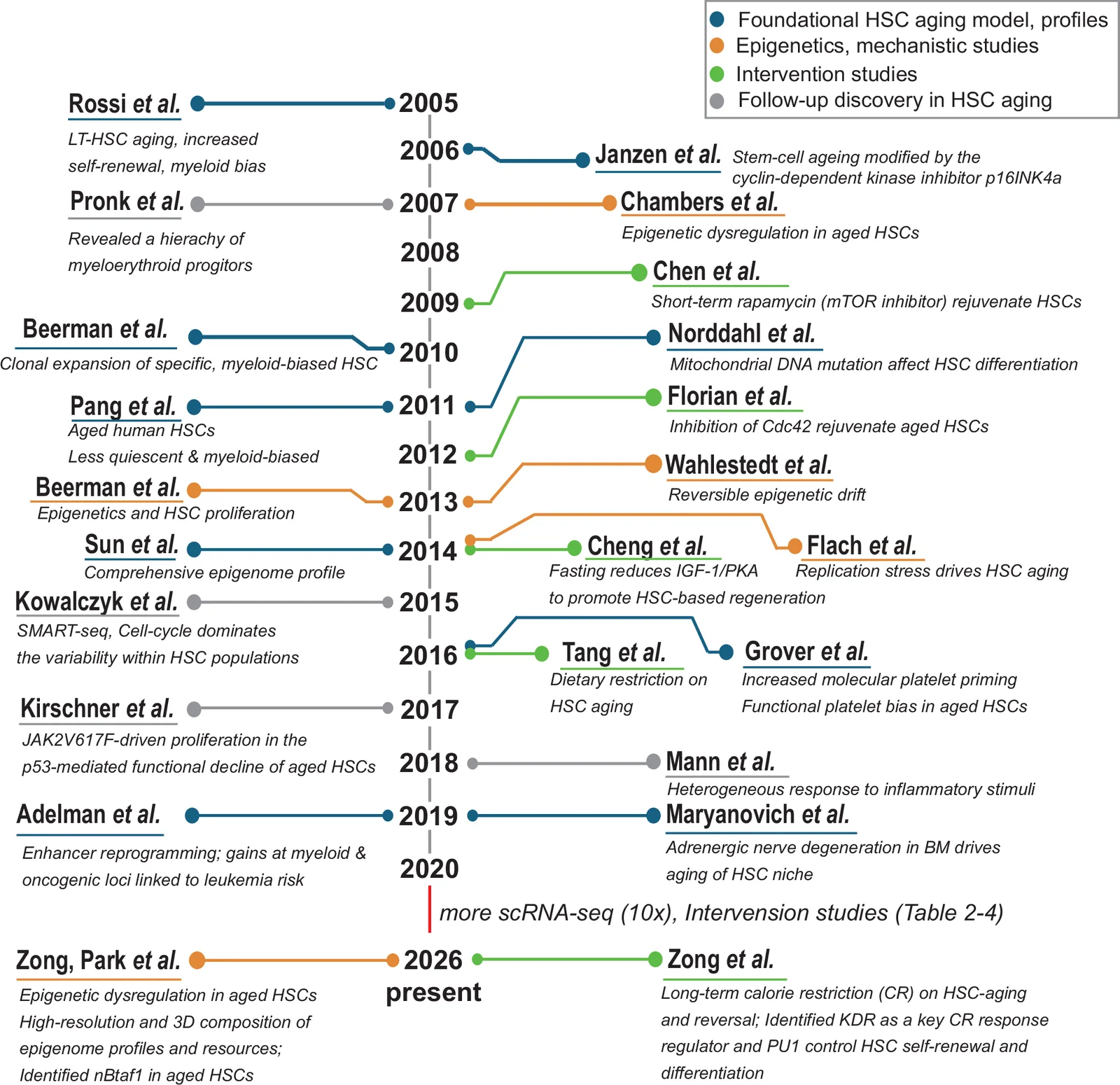
Timeline of landmark discoveries in HSC aging research. This is a chronological overview of pivotal studies that have shaped our understanding of hematopoietic stem cell (HSC) aging from 2005 to the present. Early foundational work by ref. ^14^ established the concepts of long-term (LT) HSC aging and myeloid bias. Subsequent research expanded into epigenetic dysregulation (for example, Chambers et al.^15^ and Sun et al.^5^), single-cell transcriptomics (for example, Kowalczyk et al.^65^), and the role of the bone marrow niche (for example, Maryanovich et al., 2019). Recent advancements include high-resolution 3D epigenome profiling and LT intervention studies, highlighting the continuous evolution of the field toward potential rejuvenation strategies. BM, bone marrow; scRNA-seq, single-cell RNA sequencing.

A historical timeline of key transcription and epigenomic profiling studies on HSC aging is presented in Fig. 2. Progressing from early microarray analyses^14,15,24^ that revealed myeloid skewing, stress responses, and epigenetic changes, these influential public datasets and pioneering findings demonstrated proliferation-dependent DNA methylation alteration^25,26^ and broadened H3K4me3 peaks^5^. The transition to bulk RNA-seq offered higher resolution and quantitative depth and was often integrated with epigenomics^3,21,27,28^. Many foundational studies were performed with murine models, but their findings overlap with human studies^3,29^. Notable examples include human-focussed profiling that replicated increased HSC frequency and myeloid bias with age^29^. Table 1 presents some foundational publications on HSC aging studies with public datasets (with GEO accessions) in rigorously purified HSC populations. These publications were selected for historical impact, citation frequency, and reusability in meta-analyses. These and other datasets were used to identify murine HSC aging signature genes based on consistency and reproducibility between experiments (2007–2014)^30^. Using microarray (*N* = 5) and bulk RNA-seq (*N* = 3) approaches as well as drawing upon scRNA-seq datasets (*N* = 4), more than 200 murine genes have been identified as exhibiting age-associated expression change, with top ranked genes including *Selp*, *Mt1*, *Nupr1*, *Clec1a*, and *Plscr2* (ref. ^30^) (https://agingsignature.webhosting.rug.nl/publications).

**Table 1:** Key transcription and epigenomic profiling studies.

GEO accession	Platform	Species	Publication (year; PMID)	Key comparison	Key findings
–	Microarray	Mouse	Rossi et al. (^14^; PMID: 15967997)	Young, mid, old mice	LT-HSC aging accompanied by cell-autonomous changes, including increased stem cell self-renewal, differential capacity to generate committed myeloid and lymphoid progenitors, and diminished lymphoid potential
GSE6503	Microarray	Mouse	Chambers et al. (^15^; PMID: 17676974)	Young (2 months) vs aged (21 months) HSCs	Functional decline of HSCs despite their increased numbers; upregulated stress/inflammation genes, downregulated chromatin regulators
GSE32719	Microarray	Human	Pang et al. (^29^; PMID: 22123971)	Young vs aged human bone marrow HSCs	Increased HSC frequency, reduced quiescence, and myeloid bias in aged human HSCs; early cell-intrinsic evidence in humans
GSE44117	Bisulfite-seq (RRBS)	Mouse	Beerman et al. (^16^; PMID: 23415915)	Quiescent vs serially transplanted (proliferated) HSCs	Proliferation history drives age-like DNA methylation changes at differentiation loci, linking replicative stress to functional aging phenotypes
GSE47817	Bulk RNA-seq, ChIP-seq, bisulfite-seq	Mouse	Sun et al. (^5^; PMID: 24792119)	Young (2–3 months) vs aged (20–24 months) LT-HSCs	Upregulation of self-renewal and rRNA genes in aged HSCs; myeloid bias via epigenetic silencing of differentiation loci
GSE100428	Bulk RNA-seq	Mouse	Mann et al. (^4^; PMID: 30540934)	Young vs aged LT-HSCs ± inflammation	Heterogeneous myeloid-primed responses to stress in aged HSCs
GSE104406	Bulk RNA-seq, scRNA-seq	Human	Adelman et al. (^3^; PMID: 31085557)	Young (18–30 years) vs aged (65+ years) CD34^+^ HSCs	Enhancer reprogramming gains at myeloid and oncogenic loci linked to leukemia risk

*ChIP-seq* chromatin immunoprecipitation sequencing, *LT-HSC* long-term hematopoietic stem cell, *PMID* PubMed Unique Identifier, *RRBS* reduced representation bisulfite sequencing, *scRNA-seq* single-cell R*NA* sequencing.

As the top ranked gene associated with HSC aging, *Selp* encodes P-selectin, a crucial cell adhesion protein (also known as CD62P or GMP-140) found in platelets and endothelial cells. It has been established that P-selectin is essential for inflammation and blood clotting through its mediation in the initial binding and rolling of white blood cells (leukocytes) and platelets to blood vessel walls. *Selp* gene expression has been found to be consistently upregulated in aged HSCs across numerous independent studies. Recent functional studies have also demonstrated that P-selectin is not merely a biomarker but has a critical functional role in driving HSC aging. For example, Yang et al.^31^ demonstrated that aberrant engagement of P-selectin drives HSC aging in mice, with the P-selectin-PSGL-1 axis serving as a pivotal regulator of HSC regeneration. Similarly, He et al.^32^ identified P-selectin as a key regulator of HSC function under aging and inflammatory stress, showing that P-selectin overexpression impairs HSC homeostasis via inflammatory receptor-mediated proliferation and differentiation.

These research efforts are complemented by initial epigenomic work identifying DNA methylation alteration associated with functional decline^5,25^ along with initial analysis of histone alteration^5^. In the transition from microarray to bulk RNA-seq, more recent studies on chromatin accessibility changes have taken advantage of updated technologies to improve data resolution^21,27^. Similarly, although early methylation research used reduced representation bisulfite sequencing, whole-genome bisulfite sequencing has been applied more frequently as sequencing technology has become less cost prohibitive^33^.

## Single-cell resolution in HSC aging research

scRNA-seq has accelerated our understanding of HSC aging by resolving cellular heterogeneity, rare subpopulations, and aging trajectories that are obscured in bulk analyses. Recent scRNA-seq studies have identified age-specific shifts in cell cycle quiescence, platelet/myeloid priming, and clonal diversity loss (Table 2). Herault et al.^34^ produced a reference map of HSC differentiation with scRNA-seq datasets, and Ainciburu et al.^35^ reported transcriptional differences in human HSCs and progenitor cells (HSPCs) from young and old donors, highlighting heterogeneity and age-related functional decline. Montserrat-Vazquez et al.^36^ reported that single-cell profiling reveals changes in HSC transcriptomes that underlie enhanced lymphoid and regenerative capacity in serial transplantation assays. Such findings provide proof-of-concept evidence that a short systemic treatment to decrease Cdc42 activity improves the regenerative capacity of different endogenous aged stem cells in vivo^36^. Two recent human scRNA-seq studies report dynamic remodeling of HSPC states from prenatal stages to adulthood: with one identifying age-associated shifts in multipotency and lineage commitment^37^, and the other detecting a dramatic decrease in HSC clonal diversity with age, including oligoclonal structure and lineage bias in aged hematopoiesis^38^. More recently, Wang et al.^39^ performed scRNA-seq analysis with young and aged Rhesus macaque (*Macaca mulatta*) samples and reported the upregulation of GZMB expression across multiple cell types in aged monkeys.

**Table 2:** Recent single-cell RNA-seq studies on HSC aging and interventions.

GEO accession	Species	Publication (Year; PMID)	Key focus (aging/intervention)	Key findings
GSE147729	Mouse	Hérault et al., *BMC Biol*. (2021^34^; PMID: 33526011)	Aging	Developed a new reference map of HSC differentiation in young and aged mice. Identified a potential mechanism that delays the differentiation of aged HSCs and could promote the emergence of age-related hematological diseases
GSE180298	Human	Ainciburu et al., *eLife* (2023^35^; PMID: 36629404)	Aging	Identified transcriptional differences in HSPCs from young vs elderly donors. Highlights heterogeneity and age-related functional decline
GSE189161	Human	Li et al., *Nat. Methods* (2025^37^; PMID: 39639169)	Aging (developmental to adult)	Identified dynamic remodeling of HSPC states from prenatal stages to adulthood; found age-associated shifts in multipotency and lineage commitment
GSE261078	Human	Weng et al., *Nature* (2024^38^; PMID: 38253266)	Aging (clonal dynamics)	Found dramatic decrease in HSC clonal diversity with age; oligoclonal structure and lineage bias in aged hematopoiesis
GSE197070	Mouse	Montserrat-Vazquez et al., *npj Regen. Med*. (2022^36^; PMID: 36581635)	Intervention (CASIN Cdc42 inhibitor)	Detected changes in HSC transcriptome that underlie enhanced lymphoid and regenerative capacity in serial transplantation assays. Proof-of-concept evidence that a short systemic treatment to decrease Cdc42 activity improves the regenerative capacity of different endogenous aged stem cells in vivo
GSE230908	Monkey	Wang et al., *Adv. Sci*. (2026^39^; PMID: 41556329)	Young vs old comparison	Upregulation of GZMB expression across multiple cell types in aged monkeys
GSE310923	Mouse	White et al., *Aging Cell* (2026; PMID: 41603349)	Young vs old comparison	Lin-BM cells showed significant myeloid skewing in common myeloid progenitor (CMP) cells, as well as in the HSC population. Identified a unique HSC population marked by increased Vwf, Wwtr1, and Clca3a1 expression that does not exist in young HSCs, thus likely marking true aged HSCs
GSE329536 (private)	Monkey	Yanai et al. (2026; manuscript in preparation)	Young vs mid-late aged comparison	Distinct, lineage-specific aging signatures, with the most profound transcriptional alterations occurring in multipotent progenitors and classical monocytes. Mid-late aged HSCs reside in a more advanced state of differentiation with altered kinetics, providing a molecular basis for the observed age-related myeloid bias

*BM* bone marrow, *GZMB* granzyme B, *HSC* hematopoietic stem cell, *HSPC* hematopoietic stem and progenitor cell.

The precise identification of human HSCs based on immunophenotypic markers presents a significant challenge in aging studies, particularly when analyzing bulk populations. Less restrictive immunophenotypes, such as CD34^+^ CD38^−^, often co-isolate multipotent progenitor cells, thereby confounding the interpretation of age-related changes attributed solely to HSCs^40,41^. More refined marker combinations, including Lin-CD34^+^CD38^−^CD90^+^CD45RA^−^, are increasingly used to achieve a purer HSC population, enabling a more accurate assessment of intrinsic HSC aging phenotypes^42^. This heterogeneity in isolation strategies underscores the need for careful consideration of marker panels to ensure the specificity of findings in human HSC research.

## Proteome and metabolomics datasets in HSC aging studies

High-resolution proteomic and metabolomic datasets have also clarified the molecular changes occurring in HSCs during aging. A comprehensive mouse HSC proteomics resource quantified more than 5000 proteins across young adult and old adult HSCs and early progenitors, revealing increased protein diversity in aged HSCs, unique proteomic signatures, and evidence of post-transcriptional regulation that distinguishes stem cells from progenitors^43^. This dataset is also publicly available via ProteomeXchange^43^. In humans, quantitative proteomics of bone marrow HSPCs from donors spanning a wide age range identified age-associated alterations in multiple cell populations, including shifts in metabolic and signaling pathways within the HSC compartment^44^. Complementary metabolomics efforts, such as integrated metabolome and lipidome profiling of human BM HSPCs (lineage⁻CD34⁺CD38⁻), demonstrated declines in key metabolites such as choline with aging (and further in leukemia), alongside broader changes in lipid, amino acid, and nucleotide profiles in aging murine bone marrow^45,46^.

## Multi-omics integration in HSC aging studies

Multi-omics integration has markedly advanced our understanding of HSC aging by linking transcriptional changes to upstream epigenetic regulation, post-transcriptional protein dynamics, and downstream metabolic alterations, revealing causal hierarchies that single-layer analyses obscure. Early bulk-level studies integrated transcriptome profiling with epigenomics (DNA methylation, histone marks, and ATAC-seq), demonstrating that age-associated enhancer reprogramming and methylation drift at differentiation loci drive myeloid bias and self-renewal gene upregulation in both mouse and human HSCs^3,5,25^. Recent single-cell multi-omics approaches have elevated this resolution, combining transcriptomics with epigenomics (for example, scRNA-seq + scATAC-seq) in lifespan-resolved human HSC atlases to uncover subset-specific aging trajectories and heterogeneity^47^. Integrating metabolomics into multi-omics analyses remains at an early stage but has already proven to be insightful in contexts like NAD^+^ decline, linking mitochondrial dysfunction with inflammatory priming^48^. Overall, these multilayer datasets expose reversible epigenetic–metabolic nodes for rejuvenation but are limited in scope and accessibility; comprehensive portals are needed to harmonize them for meta-analyses. Our research team also has been working to generate comprehensive epigenome profiles including RNA-seq, chromatin immunoprecipitation sequencing (histone modifications, Spi1 transcription factors), ATAC-seq (chromatin accessibility), HiC (chromatin architecture), and DNA methylation datasets, which could also be incorporated into multilayer omics datasets^21,33^.

## Interventions for HSC rejuvenation

In addition to characterizing natural HSC aging, several studies have explored interventions to mitigate or reverse age-associated decline, with evidence of partial rejuvenation (for example, restored self-renewal, balanced differentiation, mitochondrial function, or improved engraftment). Table 3 summarizes HSC intervention examples, focussing on those demonstrating functional rejuvenation in aged HSCs. Researchers have found that aged HSCs have increased glucose use and higher levels of glycolysis but mitochondrial function impairment^44,49^. Metabolic interventions such as calorie restriction (CR) improve repopulation but impair lymphoid differentiation capacity^50,51^. Our research team also performed long-term CR treatment and found that lifelong CR mitigates age-associated HSC transcriptome changes, but these modifications are swiftly lost after ad libitum feeding^28^. Epigenetic profiling has identified KDR as a key regulator of the CR response and has revealed that experimental knockdown of Kdr in aged HSCs reproduces the more youthful aging transcriptome observed in lifelong CR HSCs^28^. These interventions highlight reversible aspects of HSC aging, particularly through epigenetic (PU.1 acting as an intracellular regulator of the CR response), metabolic (for example, NAD^+^ restoration alleviating mitochondrial decline)^52,53^, and niche-targeted pathways. Recently, Arif et al.^54^ discovered that v-ATPase inhibition rejuvenates stem cell functions, whereas Mejía-Ramírez et al.^55^ also found that targeting RhoA nuclear mechanoactivity rejuvenates HSCs. Although functional rejuvenation studies typically present only partially effective outcomes, they provide proof-of-concept for HSC aging therapies.

**Table 3:** Key intervention studies demonstrating potential rejuvenation of aged HSCs.

GEO accession (if available)	Intervention	Species	Publication (year; PMID)	Key findings
–	CASIN (Cdc42 inhibitor)	Mouse	Florian et al., *Cell Stem Cell* (2021^12^; PMID: 22560076)	Aged HSCs elevated Cdc42 activity; CASIN treatment restores polarity, reduces myeloid skewing, and rejuvenates long-term HSC repopulation
–	Prolonged fasting (calorie restriction (CR) mimetic)	Mouse	Cheng et al., *Cell Stem Cell* (2014; PMID: 24905167)	Prolonged fasting reduces IGF-1/PKA signaling, promotes regenerative capacity, and reverses immunosuppression in aged HSCs
–	Dietary restriction	Mouse	Tang et al., *J. Exp. Med*. (2016^50^; PMID: 33331868)	Dietary restriction enhances HSC quiescence, increases serial repopulation capacity, and reduces myeloid bias but may impair lymphoid differentiation
GSE165982	Nicotinamide riboside (NR) supplementation	Mouse	Sun et al., Nat. *Commun*. (2021^52^; PMID: 33976125)	NR treatment decreases the age-enlarged stem cell pool and shifts aged HSC functionally, metabolically, and molecularly toward the young state
GSE147662	NR supplementation	Mouse	Zong et al., *npj Aging Mech. Dis.* (2021^53^; PMID: 34548492)	NR treatment-improved lymphoid potential was maintained in competitive transplants, although the altered transcriptional priming of the stem cells toward lymphoid lineages was not sustained in the aged mice after NR removal
GSE252062	MyHSC depletion	Mouse	Ross et al., *Nature* (2024; PMID: 38538791)	Transfer of balanced HSCs results in abundant lymphoid and myeloid cells with a stable phenotype retained after secondary transfer; myeloid-biased HSCs also retain their patterns of production post-secondary transfer
GSE233989	RhoA nuclear mechanoactivity	Mouse	Mejía-Ramírez et al., *Nat. Aging* (2025; PMID: 41286464)	Inhibiting RhoA reduces tension, restores heterochromatin (e.g., H3K9me2), suppresses retrotransposons and inflammation, upregulates Klf4, and improves regenerative capacity and lymphoid/myeloid balance in transplants
–	v-ATPase inhibition	Mouse	Arif et al., *Cell Stem Cell* (2025; PMID: 41289991)	Inhibiting v-ATPase restores lysosomal integrity, reduces inflammation, and boosts old HSCs’ repopulation capacity more than eightfold ex vivo/in vivo
GSE284988	Dietary/CR	Mouse	Zong et al., *Nat. Commun*. (2026; PMID: 41720793)	Epigenetic profiling identified KDR as a key regulator of the CR response; experimental knockdown of Kdr in aged HSCs reproduced the more youthful aging transcriptome observed in lifelong CR HSCs

*HSC* hematopoietic stem cel, *IGF-1* insulin-like growth factor 1, *PKA* protein kinase A, *PMID* PubMed Unique Identifier

## Sex difference datasets in HSC aging study

Emerging evidence highlights sexual dimorphism in HSC biology, particularly during aging, with females exhibiting slower aging characteristics^56,57^. We reviewed molecular studies examining sex differences in HSC aging and summarized key findings in Table 4, incorporating datasets from our own investigations of sex-specific effects in aged HSCs (Tekin-Turhan et al., manuscript in preparation). The majority of aging signature genes have been derived from male datasets, whereas female samples in studies that include them frequently exhibit slower aging characteristics, including delayed myeloid skewing, preserved lymphoid output, and distinct responses to niche signals and hormones^56,57^ (for example, estrogen supporting proliferation without exhausting the stem cell pool, and follicle-stimulating hormone promoting expansion). Males, by contrast, often exhibit earlier or more severe declines, such as accelerated myeloid bias, anemia, and increased leukemogenic potential^57^. These patterns align with human epidemiology, including higher risks of myeloid malignancies in aged males, and underscore the importance of stratifying aging studies by sex. However, dedicated molecular datasets focussed on sex differences remain scarce relative to broader aging cohorts, where sex is typically treated as metadata rather than a primary variable of interest.

**Table 4:** Key studies on sex differences in HSC aging.

GEO accession	Species	Publication (year; PMID)	Key focus	Key findings
GSE27686	Mouse	Wahlestedt et al., *Nat. Commun* (2017; PMID: 28224997)	Both male and female mice were used for experiments	Accumulating mitochondrial DNA mutations drive premature hematopoietic aging phenotypes molecularly distinct from physiologic stem cell aging
GSE151333	Mouse	Young et al., *Cell Stem Cell* (2021; PMID: 33848471)	LT-HSC from young (2 months), middle-aged (12 months), and old (22 month) mice for RNA-seq (female)	Decreased local IGF-1 in the middle-aged bone marrow microenvironment induces and is indispensable for hematopoietic stem cell aging
GSE157455	Mouse	Kim et al., *Nat. Commun.* (2022; PMID: 36057685)	Young vs old female HSCs	TAZ buffers age-related loss of PU.1 activity to maintain HSC functionality. Surface protein Clca3a1 is a marker of “young-like” HSCs, even in old mice
GSE219092	Mouse	Meng et al., *Nat. Cell Biol*. (2023; PMID: 37127714)	Young vs old female HSCs	Platelet-biased HSCs show elevated levels of epigenetic platelet-lineage priming and give rise to MPP2 progenitors with molecular platelet bias. These MPP2 progenitors generate platelets with faster kinetics and through a more direct cellular pathway compared with MPP2s derived from multilineage HSCs
GSE215923 (private)	Mouse	Tekin-Turhan et al. (2026, manuscript in preparation)	Young, mid, vs old female HSCs	We uncover distinct, sex-specific trajectories of HSC and hematopoietic aging, underscore the dominant influence of the environment, and offer a resource to guide mechanistic studies and the development of sex-tailored therapeutic strategies

*HSC* hematopoietic stem cell, *IGF-1* insulin-like growth factor 1, *LT* long-term, *MPP* multipotent progenitor, *PMID* PubMed Unique Identifier, *RNA-seq* RNA sequencing

## Dataset integration challenges and strategies

Harmonizing multiple datasets across platforms and modalities remains a core challenge in genomics research including HSC aging analysis. Bulk transcriptional profiling — from early microarrays to modern RNA-seq (as summarized in Table 1) — has defined conserved signatures such as myeloid bias and self-renewal reinforcement, but batch effects, varying cell purity, and platform differences complicate cross-study comparisons. Tools such as ComBat-seq^58^, Harmony^59^, or limma’s removeBatchEffect^60^ have proven effective for meta-analyses of various transcriptome datasets, whereas multi-omics frameworks such as Multi-Omics Factor Analysis (MOFA+)^61^, tensor decomposition, or joint embedding models (for example, scVI^62^ or totalVI^63^) enable integration of transcriptomics with epigenomics (for example, DNA methylation and ATAC-seq), proteomics, and emerging metabolomics layers. However, completely removing batch effects remains a fundamental challenge and probably impossible to resolve. Thus, researchers prefer to perform a pair-wise comparison within their own studies and then compare internal results with other studies through differentially expressed gene ranking (for example, Rank–Rank Hypergeometric Overlap analysis^64^). Extending integration of multi-omics datasets to intervention cohorts and supporting sex-stratified data will be important for uncovering modifiable pathways in diet and pharmacological interventions informed by age and sex differences. Researchers could also utilize general human reference maps (for example, Azimuth) to align their own human, primate, and animal studies to such maps and investigate differences among samples.

## Incorporating single-cell and multi-omics resolution

Bulk analyses average heterogeneous signals, obscuring subpopulation dynamics revealed by scRNA-seq (Table 4). The scRNA-seq studies have identified age-specific shifts in cell cycle quiescence, platelet/myeloid priming, and clonal diversity loss, whereas multi-omics extensions (for example, paired scRNA-seq/scATAC-seq in lifespan atlases) expose subset-specific epigenetic drivers of functional decline^18,34,65^. The main limitation of current single-cell datasets is the relatively low coverage of expressed genes (in scRNA-seq) or accessible chromatin regions (in scATAC-seq) per cell, combined with the high cost of substantially increasing sequencing depth. To advance multi-omics integration, single-cell research datasets on cells from well-defined cell types corresponding to specific HSC or early progenitor populations (for example, lymphoid-primed multipotent progenitors [LMPP], granulocyte-monocyte progenitors [GMP], and erythroid-myeloid progenitors [EMP]) could be aggregated to generate pseudo-bulk profiles. These profiles could then be analyzed together with existing bulk RNA-seq datasets using established integrative methods.

## Leveraging artificial intelligence and machine learning in HSC aging research

The next frontier in HSC aging research is to consolidate publicly available multi-omics resources into a unified, well-curated framework. This would establish a foundational dataset enabling advanced, AI-driven integration and discovery. This step involves curating high-quality genomics datasets in accessible, standardized formats to facilitate integrated analyses using established pipelines. In addition, as sequencing costs decline and technologies improve coverage and depth, scRNA-seq and snATAC-seq datasets will increasingly support robust AI-driven analyses at the true single-cell level. As discussed in a recent review by ref. ^66^, AI can be leveraged to dissect regulatory networks in senescent cells, highlighting its potential in precision aging biology through single-cell multi-omics and advanced computational strategies. Trained on harmonized datasets (for example, from Tables 1 to 4), AI agents could develop “aging clocks,” predict intervention efficacy (such as virtual screening of NAD⁺ boosters), or simulate cellular trajectories in organoids. Community databases hosting pretrained models would democratize access to these tools.

**Fig. 3:**
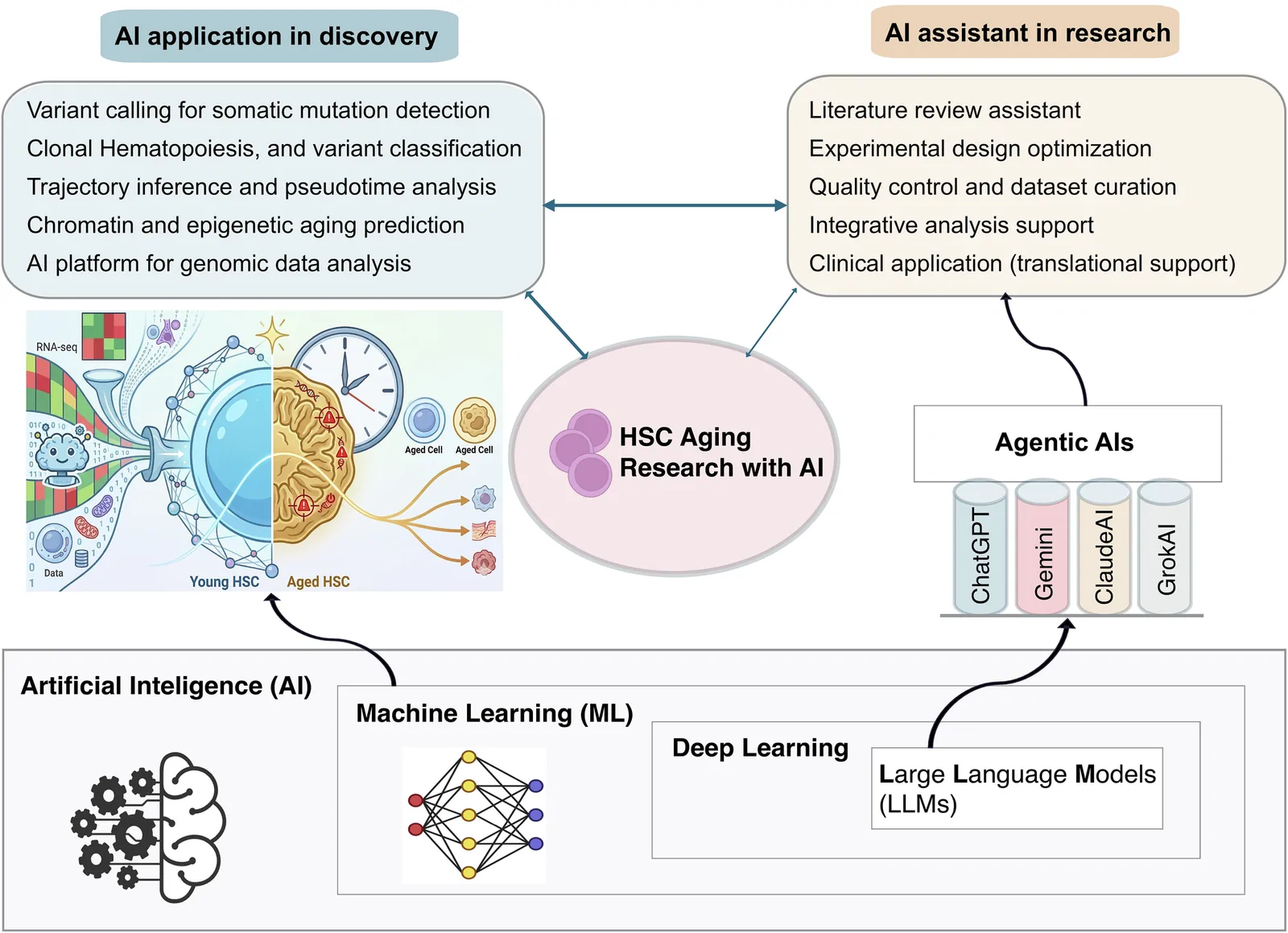
Integration of artificial intelligence in HSC aging research. This diagram outlines the multifaceted applications of AI in advancing hematopoietic stem cell (HSC) aging research. AI technologies, which encompass ML, deep learning, and LLMs, can serve dual roles. These tools can be utilized for complex genomic data analysis, including variant calling for somatic mutations, trajectory inference, pseudotime analysis, and epigenetic aging predictions. Concurrently, agentic AIs (supported by ChatGPT, Gemini, Claude, and GrokAI) can act as research assistants by streamlining the scientific workflow by assisting in literature reviews, experimental design optimization, dataset curation, and translational support. This synergistic approach accelerates both fundamental discoveries and the practical execution of research methodologies. RNA-seq, RNA-sequencing.

Table 5 lists selected commercial and open-source genomics AI tools^67–76^, chosen for their impact, adoption, dataset scale, performance in drug discovery/precision medicine, and relevance to aging/longevity research. Figure 3 illustrates the multifaceted applications of AI in advancing HSC aging research.

**Table 5:** Selected commercial and open-source AI genomics platforms/tools.

Platform/tool	Class	Key features	Primary applications	Notes/status
**Tempus**	Commercial	Multimodal AI (genomics, clinical, imaging); therapy matching and trial optimization	Precision oncology, cardiology, and rare diseases	World’s largest clinical-molecular dataset; widely partnered with pharmaceutical companies
**Deep Genomics (BigRNA)**	Commercial	RNA foundation model; predicts genetic effects on RNA; oligonucleotide design	Rare diseases, RNA therapeutics, and drug discovery	Flagship platform for genetic medicine
**SOPHiA GENETICS**	Commercial	AI for variant interpretation, clinical insights, and multimodal data analysis	Precision medicine, diagnostics, and oncology	Deployed in hundreds of hospitals globally; widely used in radiogenomics
**NVIDIA Clara, BioNeMo Framework**	Commercial/open-source	GPU-accelerated pipelines; foundation models for genomics and biology	AI foundation models for omics, protein and molecular structures, imaging, 3D anatomy, surgical robotics, and digital twins for physics-informed simulations	Provide framework (e.g., BioNeMo Framework) for AI implementation and support Python libraries
**Alpha Genome**	Commercial	Comprehensive multimodal prediction: AlphaGenome predicts 11 different molecular modalities (properties) simultaneously	Analyze and predict the function of DNA sequences, e.g., non-coding regulatory sites	A unified DNA sequence model; 1 Mb of DNA input can sequence and predict thousands of functional genomic tracks up to single-base-pair resolution across diverse modalities
**DeepVariant**	Open source	Deep learning-based tool developed by Google for identifying genetic variants	Genetic variants (e.g., SNVs and InDels) using sequencing datasets	Automatically produces filtered variants without post-calling refinement and supports both short-read and long-read sequencing technologies; used in the UK Biobank WES consortium
**Evo 2 (Arc Institute)**	Open source	Largest genomic foundation model (40B params); processes 1M+ nucleotides; supports genome design.	Mutation prediction, protein design, synthetic biology	Fully open weights/code/data; trained on 128k+ genomes across all life domains
**Nucleotide Transformer (InstaDeep)**	Open source	Transformer models for DNA/RNA; variant effects; and regulatory prediction	Genomics benchmarks, promoter/enhancer tasks	Integrating information from 3,202 human genomes and 850 genomes from diverse species
**HyenaDNA**	Open source	HyenaDNA scales subquadratically in sequence length, uses single-nucleotide tokens. Long-context model (1M+ tokens)	Sequence classification and expression prediction. Efficient for whole-genome tasks	Training up to 160× faster than Transformer
**DNABERT-2**	Open source	BERT-style pretraining on multispecies DNA; embeddings for downstream tasks	Variant effects, gene essentiality, regulatory elements	Large-scale multispecies genome that achieves the state-of-the-art performance
**ClockBase Agent**	Open source	AI agents with 40+ aging clocks; tool for re-analyzing public datasets for interventions	Aging biomarker discovery, longevity drug screening	Processes millions of public samples; DNA methylation-based aging marker
**MetaCH**	Open source	Open-source machine-learning framework that classifies variants in cfDNA from plasma-only samples as clonal hematopoiesis (CH) or tumor origin	Accurate detection of somatic variants of CH origin in plasma samples	Detection of CH
**ChromAgeNet**	Open source	Deep learning approach based on convolutional neural networks (CNNs) applied to 3D microscope images	Aging biomarker discovery, chromatin architecture	Trained algorithm on 3D microscope images of DAPI-stained HSC nuclei; learns changes in the spatial features of chromatin architecture during natural aging of HSCs
**scAge**	Open source	Gated multihead attention (GMA) neural network	Epigenetic age profiling at single-cell resolution	Recapitulates the chronological age of tissues while uncovering heterogeneity among cells

*AI* artificial intelligence, *cfDNA* cell-free *DNA*, *DAPI* 4',6-diamidino-2-phenylindole, *GPU* graphics processing unit, *HSC* hematopoietic stem cell, *SNV* single-nucleotide variant.

## AI-enhanced variant calling for somatic mutation detection

Accurate detection of somatic mutations that accumulate with age is crucial for understanding the genomic basis of HSC aging. These are somatic variants in hematopoietic cells acquired over a lifetime^77^, often affecting genes associated with aging, and those commonly altered in hematological malignancies^78^. AI-powered variant callers have demonstrated superior performance compared with traditional statistical methods, offering higher accuracy and sensitivity for detecting low-frequency variants in heterogeneous HSC populations. For example, DeepVariant^68^, developed by Google Health, uses deep convolutional neural networks to analyze pileup image tensors of aligned reads, achieving higher accuracy compared with traditional tools such as GATK^79^, FreeBayes^80^, and SAMTools^81^. DeepVariant automatically produces filtered variants without post-calling refinement and supports both short-read and long-read sequencing technologies. It has been used in large-scale studies including the UK Biobank WES consortium (500,000 individuals)^82^. The improved accuracy reduces false positives that can confound aging signatures and enables detection of low-frequency variants that may represent early clonal expansions. As single-cell sequencing of aged HSCs becomes more common, AI variant callers will be essential for managing the increased noise and lower coverage inherent in single-cell data.

## AI for clonal hematopoiesis detection and variant classification

One of the most specific applications of AI in hematopoietic aging is the detection and classification of clonal hematopoiesis (CH) variants. CH variants comprise more than 75% of cell-free DNA (cfDNA) variants in individuals without cancer and sometimes more than 50% of cfDNA variants in patients with cancer^83^, making accurate CH variant detection critical for liquid biopsy applications and age-related disease monitoring. MetaCH (Metaclassifier for Clonal Hematopoiesis detection), open-source machine-learning framework, classifies variants in cfDNA from plasma-only samples as CH and/or oncogenic without requiring matched white blood cell sequencing^74^. MetaCH uses a three-stage architecture: (1) the Mutational Enrichment Toolkit (METk) generates variant embeddings, gene embeddings, and functional prediction scores using self-supervised learning; (2) base classifiers distinguish CH-oncogenic from CH-non-oncogenic variants using pan-cancer datasets; and (3) a metaclassifier integrates these scores into a single-variant origin prediction. AI-based CH detection enables more accurate identification of age-related clonal variants versus disease-related mutations, facilitating studies of clonal dynamics, evolutionary trajectories, and the relationship between CH and functional HSC decline.

## Machine learning for trajectory inference and pseudotime analysis

Understanding how HSCs transition from young to aged states requires computational methods that can order cells along aging trajectories and infer differentiation paths. Machine-learning-based trajectory inference methods have become essential tools for analyzing single-cell data in aging contexts. Several advanced methods are particularly relevant for HSC aging: VITAE uses deep learning with mixture priors for joint trajectory inference across multiple datasets, enabling comparison of aging trajectories across conditions^84^. Lamian provides a statistical framework for differential multisample pseudotime analysis, enabling rigorous comparison of trajectories between young and aged HSCs^85^. FateNet integrates dynamical systems with deep learning to predict cell fate decisions, applicable to modeling HSC differentiation trajectories and how they change with age^86^. These methods enable researchers to map aging trajectories from young to old HSCs, order cells along an aging continuum using pseudotime, predict differentiation outcomes of aged HSCs, compare trajectories between intervention conditions, and identify subpopulations with distinct aging trajectories. Artificial neural networks have also been successfully applied to annotate hematopoietic progenitor and HSC-like cells derived from human pluripotent stem cells, demonstrating the power of machine learning for unbiased cell type identification from gene expression patterns^87^.

## Deep learning for chromatin-based aging prediction

Epigenetic changes that alter chromatin architecture are suggested to contribute significantly to HSC aging. ChromAgeNet, an interpretable deep learning framework based on convolutional neural networks, predicts HSC aging from 3D chromatin organization in 4′,6-diamidino-2-phenylindole-stained microscopy images^75^. This approach demonstrates that changes in chromatin organization can be modeled via machine learning to predict cellular aging without requiring molecular profiling. ChromAgeNet learns aging-associated signatures of chromatin changes directly from nuclear morphology, providing a non-invasive and cost-effective method for aging assessment. The framework is interpretable, allowing researchers to understand which chromatin features contribute most to aging predictions. This connects structural chromatin changes to functional aging phenotypes and provides mechanistic insights into epigenetic aging processes. Related work has shown that the nuclear protein lamin A/C regulates epigenetic and chromatin architecture changes in aged HSCs^88^, and that epigenetic traits inscribed in chromatin accessibility drive aged HSC phenotypes^27^. Additionally, aging-disturbed FUS phase transition impairs HSC function by altering chromatin structure^89^. These studies collectively establish chromatin architecture as both a biomarker and a functional driver of HSC aging, making chromatin-based AI models particularly valuable for intervention studies in which chromatin remodeling may lead to rejuvenation.

## Deep aging clocks for biological age estimation

Deep learning has revolutionized the development of aging clocks — predictive models that estimate biological age from molecular or physiological data^90^. Deep aging clocks leverage neural networks to analyze diverse data types including epigenetics (DNA methylation), transcriptomics, metabolomics, microbiome composition, and imaging data^91–95^. These models can predict chronological age with high accuracy and, more importantly, identify individuals whose biological age deviates from chronological age, indicating accelerated or decelerated aging. For hematopoietic aging specifically, several approaches show promise. A hematology-based clock derived from the Study of Longitudinal Aging in Mice uses deep neural networks to estimate biological age from blood parameters, with functional changes in hematopoiesis serving as strong age predictors^96^. DeepMAge uses deep learning on DNA methylation data to create aging clocks that outperform traditional linear models^97^. The ClockBase platform represents an emerging resource that provides AI agents with access to more than 40 aging clocks^73^ and enables re-analysis of public datasets for aging biomarker discovery and longevity drug screening. In HSC aging research, existing epigenetic clocks can quantify the approximate biological age of HSC populations and predict functional decline before it becomes phenotypically apparent. However, these clocks were primarily developed and trained on terminally differentiated tissues (for example, peripheral blood mononuclear cells, liver, lung, and brain). To fully leverage this approach in stem cell biology, more comprehensive HSC-specific aging clocks are needed. Ultimately, such clocks would enable us to evaluate the efficacy of rejuvenation interventions and stratify HSCs by biological age for mechanistic studies. Integrating multiple modalities — epigenetic, transcriptomic, and proteomic aging clocks — may provide the most robust and accurate assessment of HSC aging status.

## AI platforms for genomic data analysis and interpretation

Several commercial and open-source AI platforms are transforming how genomic data are analyzed and interpreted in aging research. These platforms offer advanced tools for pattern recognition, automated analysis, and clinically actionable insights from complex genomic datasets. Commercial platforms include Tempus, which provides multimodal AI integrating genomics, clinical data, and imaging for precision medicine applications; Deep Genomics (BigRNA), which features RNA foundation models that predict tissue-specific regulatory mechanisms of RNA expression, the binding sites of proteins and microRNAs, and the effects of RNA variants and molecular therapeutics; and SOPHiA GENETICS, which applies AI for variant interpretation and multimodal data analysis and is deployed in hundreds of hospitals globally. NVIDIA Clara provides commercial and open-source graphics processing unit-accelerated genomic pipelines and foundation models along with AI tools (for example, BioNeMo). Open-source platforms include Evo 2 from the Arc Institute, the largest genomic foundation model with 40 billion parameters trained on 128,000+ genomes across all life domains; HyenaDNA, a long-context model that handles more than 1 million tokens for whole-genome tasks; and DNABERT-2, which provides BERT-style pretraining on multispecies DNA for variant effects and regulatory element prediction. These platforms accelerate analysis of large genomic datasets, identify genetic variations with enhanced accuracy, transform raw data into actionable insights, simplify complex genomic workflows, and provide clinically actionable interpretations.

## Building a next-generation resource for the HSC community

Mutant mouse models offer causal mechanistic validation absent in human observational data, whereas induced pluripotent stem cell-derived bone marrow organoids enable human-relevant in vitro aging (via serial passaging or stress induction) and genetic perturbation. Overlaying multi-omics from mutants onto organoid datasets bridges species gaps for drug validation. To achieve this integration, high-quality omics data — paired with comprehensive metadata, particularly from translational studies — are critically needed. Larger cohort studies that generate high-quality omics datasets from aged human HSCs should be prioritized.

Additionally, although HSC transplantation is a standard treatment for cancers such as leukemia, successful bone marrow engraftment requires ablation of endogenous stem cells, and further optimization of aged HSC therapies is needed before broader clinical application. Access to more high-quality omics data, detailed clinical metadata, and collaboration with experienced clinicians could accelerate translational advances in HSC aging research.

**Fig. 4:**
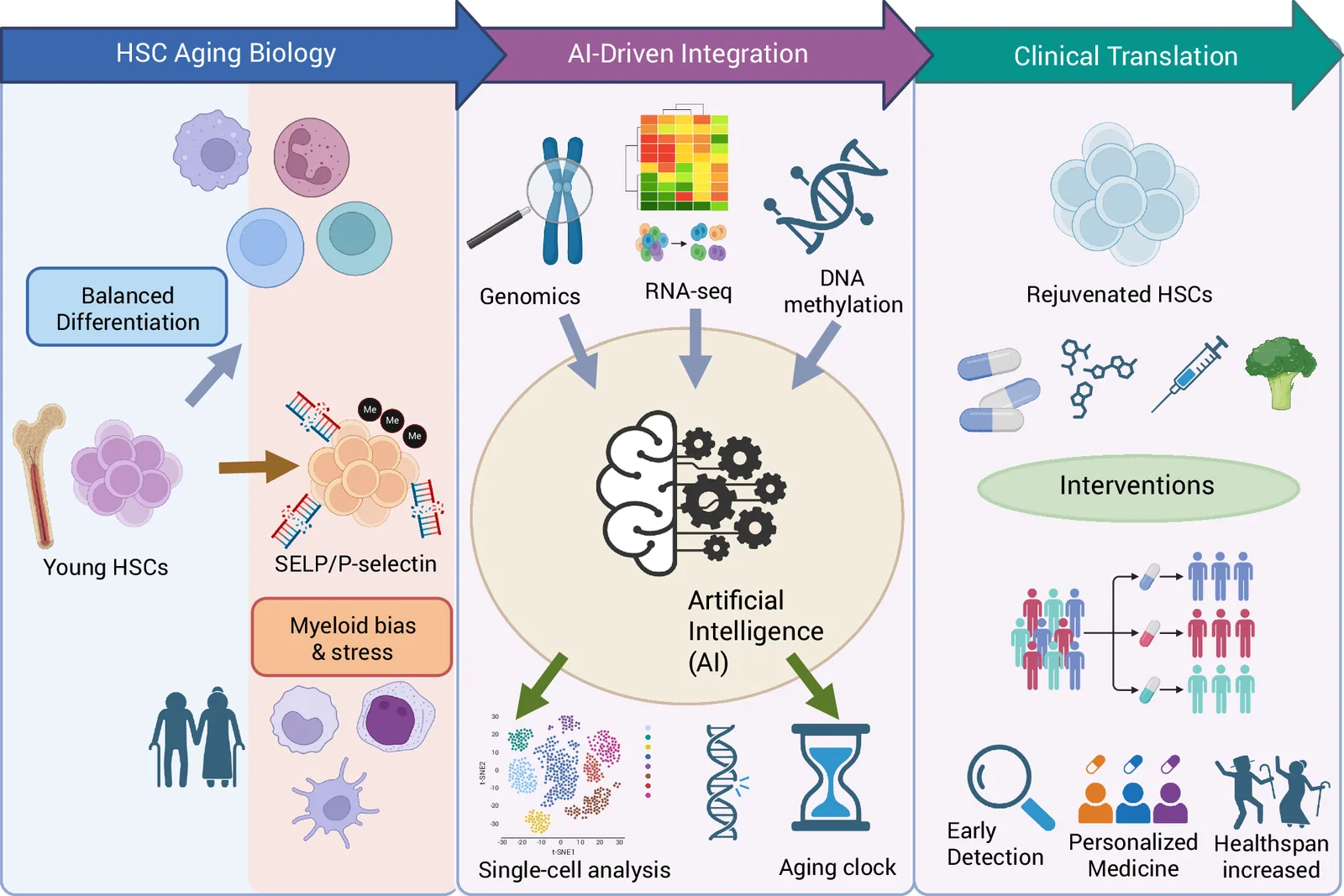
From HSC aging biology to clinical translation via AI-driven integration. This conceptual framework demonstrates how AI can bridge the gap between basic hematopoietic stem cell (HSC) aging biology research and clinical applications. The left panel depicts the biological transition from young HSCs with balanced differentiation to aged HSCs characterized by myeloid bias, stress, and specific markers such as SELP/P-selectin. The center panel highlights how multi-omics data (genomics, transcriptome, and DNA methylation) can be integrated using machine learning and AI algorithms to perform high-dimensional single-cell analysis and develop predictive aging clocks. Then as illustrated in the right panel, these AI-derived insights can inform interventions (for example, pharmacological and dietary) to rejuvenate HSCs and in turn increase the health span of aging populations. RNA-seq, RNA sequencing.

Figure 4 visualizes the trajectory of how AI-driven HSC aging research can support clinical translation. Bulk RNA-seq has illuminated core transcriptional hallmarks of HSC aging, yet disparate datasets limit deeper insights. By integrating bulk RNA-seq with multi-omics, single-cell datasets, AI-driven analyses, and translational models — from mutant mice to human organoids — a comprehensive database would catalyze breakthroughs. Starting with the key studies reviewed earlier and expanding inclusively, this living resource would empower meta-analyses, hypothesis generation, and therapies targeting age-related hematopoietic decline, fostering precision medicine for aging and blood disorders. We also recommend the broader application of AI tools to integrate multi-omics datasets in aging research.

## Applying agentic AI to hematopoietic stem cell aging research

Key ideas

- Literature review assistance. Researchers could use AI-powered tools to systematically retrieve and analyze historical and current literature on HSC aging research. The AI can identify key genes (for example, through mutant versus wild-type comparisons), highlight emerging patterns, and suggest potential future research directions. To broaden insights, such tools could also explore trends in other stem cell fields (for example, neural or muscle stem cells) and propose how those findings might translate to HSC aging. In addition, researchers can also utilize NotebookLM to create scientific slides, podcasts, and summaries from the published papers to further facilitate scientific communication.
- Experimental design optimization. AI can assist in designing experiments by suggesting optimal parameters, controls, and entire experimental workflows. This includes AI-powered CRISPR design tools for optimizing guide-RNA design for aging-related gene studies, predictive modeling to simulate potential experimental outcomes, power analysis to determine optimal sample sizes, and experimental prioritization based on predicted information gain. For HSC aging research, AI can suggest optimal timepoints for aging studies, recommend appropriate controls for intervention experiments, design efficient screening strategies for rejuvenation compounds, and optimize single-cell sequencing depth and coverage.
- Quality control (QC) and dataset curation. With the rapid growth of single-cell and bulk sequencing data, manual QC and integration become bottlenecks for researchers. Dedicated AI tools could flag low-quality samples, normalize datasets, summarize key findings, and highlight the most reliable and recent resources, facilitating easier meta-analysis. For example, we could develop a customized LLM model that is trained on multiple QC criteria and then, based on the model, create a domain-specific AI agent capable of assessing new sequencing datasets.
- Integrative analysis support. Scientists could use an AI platform that integrates new experimental datasets with precurated, quality-controlled historical data. Such a platform could perform robust meta-analyses to uncover hidden patterns and novel insights that individual studies might miss. The platform could automatically test multiple machine-learning approaches (for example, clustering, differential expression, trajectory inference, or predictive modeling) to identify the most robust and biologically meaningful results. For example, Swanson et al.^98^ utilized Virtual Lab to create multiple role-based AI agents that discovered SARS-CoV-2 antibodies.
- Clinical application. Gerontologists and other clinicians could use AI tools to translate complex HSC aging research findings into actionable clinical insights. This could include predicting age-related hematopoietic decline, identifying therapeutic targets, supporting personalized interventions to improve immune function and blood regeneration in aging patients, and risk stratification for age-related hematological diseases. AI could be used to integrate patient clinical data with individual patient genomic profiles, predict response to rejuvenation interventions, identify individuals at high risk for clonal hematopoiesis progression, and recommend personalized treatment strategies. For example, machine-learning models could predict the presence of high-risk clonal hematopoiesis from routine blood counts, enabling early intervention.

## Limitations of AI in HSC aging research

Although AI offers many workflow enhancements in HSC aging research, it is crucial to address its constraints to consider the evolving role of the bench scientist. A primary obstacle remains the acquisition of high-quality data. HSCs are a rare population, and their isolation from murine models or human donors is a labor-intensive and time-consuming process. The available limited data in turn narrow the quality of AI-facilitated analyses.

In addition, AI still requires a “human in the loop” to develop the initial experimental design and devise workflows that may or may not include AI assistance. AI-assisted workflows still require human supervision to ensure proper implementation. Also crucially, AI draws on probability patterns that, without human guidance, are not conducive to generating creative connections.

As AI continues to be integrated into scientific research, HSC researchers will be able to focus more on domain experts and hypothesis drivers. The future of the field lies in a synergistic partnership in which the HSC biologist guides the application of AI, asking critical biological questions that computational models can help explore.
